# A Novel Method for Detecting the Two-Degrees-of-Freedom Angular Displacement of a Spherical Pair, Based on a Capacitive Sensor

**DOI:** 10.3390/s22093437

**Published:** 2022-04-30

**Authors:** Shengqi Yang, Yulei Xu, Yongsen Xu, Tianxiang Ma, Hao Wang, Jinghua Hou, Dachuan Liu, Honghai Shen

**Affiliations:** 1 Key Laboratory of Airborne Optical Imaging and Measurement, Changchun Institute of Optics, Fine Mechanics and Physics, Chinese Academy of Sciences, Changchun 130033, China; y921945329@163.com (S.Y.); 13844053159@163.com (Y.X.); flykatecn@163.com (T.M.); wanghao7600@163.com (H.W.); shenhh@ciomp.ac.cn (H.S.); 2 University of Chinese Academy of Sciences, Beijing 100049, China; 3 Jiuquan Satellite Launch Centre, Jiuquan 732750, China; hjh20jd@163.com (J.H.); ldc1995529@163.com (D.L.)

**Keywords:** spherical motion pair, capacitive angular sensor, 2-DOF angular displacement measurement, four-quadrant differential structure

## Abstract

The spherical pair has an important role in the inner frame of the stabilization mechanism of the aviation optoelectronic pod. However, its two-degrees-of-freedom (2-DOF) angular displacement signal is difficult to detect, seriously restricting its application in aviation optoelectronic pods. Therefore, this study proposes a new method to measure a spherical pair’s 2-DOF angular displacement using a spherical capacitive sensor. The capacitive sensor presented by this method realizes the measurement of the 2-DOF angular displacement of the spherical pair by integrating the spherical electrode groups in the ball head and the ball socket of the spherical pair. First, based on the geometric structure of the spherical pair, the structure of the capacitive sensor is designed, and the mathematical model for the capacitive sensor is deduced. Then, the sensor’s output capacitance, in different directions, is simulated by Ansoft Maxwell software. Finally, an experiment device is built for the measurement experiments. The simulation analysis and experimental results show that the spherical capacitive sensor has an approximately linear output in different directions, and the measured output capacitance is as high as 89.7% of the theoretical value. Compared with the existing sensors that measure the 2-DOF angular displacement signal of the ball pair, the sensor proposed in this study has an integrated structure, which can be integrated into the spherical pair. That makes it possible to apply the spherical pair to the inner frame of the aviation optoelectronic pod.

## 1. Introduction

The aviation optoelectronic pod adopts a two-axis, four-frame, universal rotation mechanism to achieve high-precision and stable target tracking. The primary function of the outer frame is to rotate in a large range and to isolate wind resistance interference. The inner frame can achieve precise inertial stabilization within a small angle range of $\pm 4^{\circ }$, which ensures the stable tracking function of the entire system [1]. The traditional inner frame structure mainly adopts an orthogonal frame. Due to its large size and long transmission path, the inner frame needs sufficient rigidity to ensure its control bandwidth. That will cause the inner frame to occupy a larger space and reduce the space ratio of the optical imaging system. While it is compared with the orthogonal frame, the spherical pair has significant technical advantages, such as a small size, more freedom of movement, and a short transmission path. If the orthogonal frame is replaced by a spherical pair in the optoelectronic pod, the space occupied by the frame will reduce significantly. That will enhance the space ratio of the optical imaging system, which is essential for improving the detection performance of the aviation optoelectronic pod.

The accurate detection of the two-degrees-of-freedom (2-DOF) angular displacement signal of the spherical pair is one of the technical difficulties limiting its application and development. Therefore, the precise measurement of the 2-DOF angular displacement of the spherical pair is significant for promoting its application in aviation optoelectronic pods.

At present, the optical detection method can be used to detect the angular displacement signal of the spherical pair. The method detects the angular displacement signal of the spherical surface by detecting the change in the surface image characteristics caused by the rotation of the spherical pair by an optical sensor or a visual sensor. However, the optical detection method has a complex optical system, and the measurement resolution is restricted by the degree of refinement of the surface image features [2,3,4].

Based on the theory of magnetic effects, W. Wang and J. Wang proposed an angle measurement technology, which uses sensor arrays to measure the spatial magnetic field changes in permanent magnets in a spherical pair to achieve angular displacement measurements [5]. This method has a good anti-interference ability, but it is difficult to detect weak signal changes in the magnetic field. It is unsuitable for precise angle measurement. Therefore, both Shaohui Foong’s team and Penghao Hu’s team used artificial intelligence algorithms to improve the magnetic effect method [6,7,8,9,10,11]. After the improvement, the measurement accuracy and resolution were significantly improved.

To further improve the angular displacement detection, Penghao Hu’s team proposed a method based on eddy current sensors. This method uses eddy current sensors to measure changes in the shape of spherical secondary metal surfaces to achieve angular displacement detection [12]. Compared with the angular displacement measurement technology of magnetic effects, their technology overcomes the technical defects of the magnetic field signal jump and low measurement resolution. However, it has high requirements for the position distribution of eddy current sensors.

The above-mentioned angular displacement detection technology has a complex structure and error superposition introduced by multiple sensor units. They cannot be integrated into the spherical pair to measure its angular displacement signal, limiting the spherical pair’s application in the inner frame structure of aviation optoelectronic pods. Therefore, designing an integrated sensor with a spherical pair structure is significant for promoting the ball pairs’ application in aviation optoelectronic pods.

The capacitive sensor has the advantages of high accuracy, a low cost, and a simple structure, which means it can be used in precision measurement fields, such as spherical pairs, CNC machine tools, and non-contact force–distance measurement [12,13,14,15,16]. Therefore, in this study, we designed a capacitive sensor with an integrated structure, which comprises an upper electrode and a lower spherical electrode, forming a four-quadrant differential setup. By analyzing the movement characteristics of the electrodes, a 2-DOF angular displacement calculation and decoupling model were established. A simulation setup and an experimental device were built. The simulation and theoretical values were compared to verify the correctness of the calculation and the decoupling model. The experimental results showed that the proposed capacitive sensor could detect the angular 2-DOF of a spherical pair.

## 2. Measurement Principle

In this chapter, we propose a capacitive sensor with a spherical pair structure, which is based on the principle of variable area capacitive measurement. The functional relationship between the 2-DOF angular displacement signal of the spherical capacitive sensor and the theoretical output capacitance of the spherical capacitive sensor is deduced. We propose the decoupling and calculation methods of the 2-DOF angular displacement signal by analyzing the mathematical model established.

### 2.1. The Structure Model of the Spherical Capacitive Sensor

**Figure 1:**
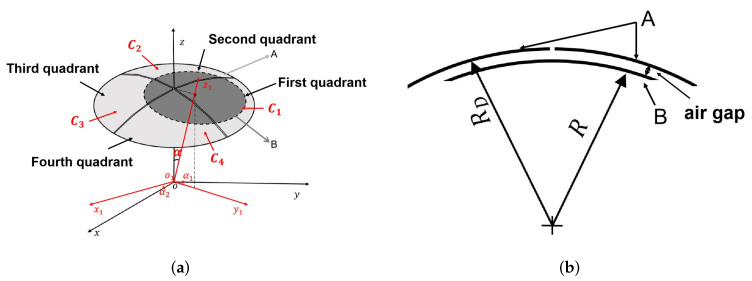
The 2-DOF angular displacement calculation model: (**a**) The schematic of the spherical capacitive sensor, (**b**) The schematic diagram of the cross-sectional of the spherical capacitive sensor.

As shown in Figure 1, to measure the 2-DOF angular displacement signal of the spherical pair, a capacitive sensor, based on the spherical differential four-quadrant setup, was designed. The capacitive sensor consists of a spherical driving electrode (A) and a spherical sensing electrode (B). The driving electrode and the sensing electrode are concentrically distributed. The concave surface of the driving electrode is divided into four equal-area parts, forming four-quadrant parts by insulating strips. The driving electrode’s four-quadrant parts, and the sensing electrode, form four measuring capacitors denoted as C1, C2, C3, and C4. At the initial position, the area between the four-quadrant parts of the driving electrode and the sensing electrode are equal, and the four-quadrant differential capacitance is in a balanced state. The sensor’s output differential capacitance signals $\Delta C_{13}$ and $\Delta C_{24}$ are zero. When the sensor is rotated along the spatial angle, the four-quadrant differential capacitors are unbalanced. Then, the sensor output’s capacitance signals are $\Delta C_{13}$ and $\Delta C_{24}$. By detecting the capacitance $\Delta C_{13}$ and $\Delta C_{24}$, a spherical pair’s 2-DOF angular displacement signal is calculated.

### 2.2. 2-DOF Angular Displacement Signal Measurement

As is shown in Figure 1, the measurement of the 2-DOF angular displacement signal of a spherical pair is equivalent to detecting the angular displacement signal component $\alpha_{1}$ of the sensing electrode around the X-axis and the angular displacement component $\alpha_{2}$ of the sensing electrode around the Y-axis. When the sensing electrode enters the first quadrant, without considering the edge effect, the expressions of the differential output capacitances $C_{13}$ and $C_{24}$ of the capacitive sensor are shown in Equations (1) and (2), respectively, where $C_{13}$ is the differential output capacitance of the first and third quadrants, and $C_{24}$ is the differential output capacitance of the second and fourth quadrants:

(1) $$\Delta C_{13}=\frac{\epsilon \cdot \Delta A_{13}}{d}$$

(2) $$\Delta C_{24}=\frac{\epsilon \cdot \Delta A_{24}}{d}$$

where ϵ is the air dielectric constant, $\Delta A_{13}$ is the differential area of the first and third quadrants, $\Delta A_{24}$ is the differential area of the second and fourth quadrants, and *d* is the gap between the two electrodes.

The expressions of the differential areas $\Delta A_{13}$ and $\Delta A_{24}$ are obtained by integrating the areas between the capacitors $C_{1}$, $C_{3}$ and $C_{2}$, $C_{4}$.

(3) $$\Delta A_{13}=H\cdot [\left(arcsin\left(\frac{R}{r}\alpha_{1}\right)+arcsin\left(\frac{R}{r}\alpha_{2}\right)\right],$$

(4) $$\Delta A_{24}=-H\cdot [\left(arcsin\left(\frac{R}{r}\alpha_{1}\right)-arcsin\left(\frac{R}{r}\alpha_{2}\right)\right].$$

where *H* is $-Rr(\pi -2arccos\frac{r}{R})$.

In this study, our measurement object is the small-range angular displacement of the inner frame of the aviation optoelectronic pod, and its 2-DOF angular displacement range is $\pm 4^{\circ }$, so that the term $\frac{R}{r}\alpha_{2}$ is a small value. Therefore, we use the Taylor series expansion to expand Equations (3) and (4), neglecting the higher-order terms of Equations (3) and (4), to simply $\Delta C_{13}$ and $\Delta C_{24}$:

(5) $$\Delta C_{13}=\frac{\epsilon HR}{rd}\cdot \left(\alpha_{1}+\alpha_{2}\right),$$

(6) $$\Delta C_{24}=-\frac{\epsilon HR}{rd}\cdot \left(\alpha_{1}-\alpha_{2}\right).$$

According to Equations (3)–(6), we can derive the differential output capacitance expressions for the sensing electrode moving into the remaining three quadrants:

(7) $$\Delta C_{13}=\left\{\begin{array}{ccc} \frac{\epsilon HR}{rd}\cdot \left(\alpha_{1}+\alpha_{2}\right) & , & First\;Quadrant,\\ \frac{\epsilon HR}{rd}\cdot \left(\alpha_{1}-\alpha_{2}\right) & , & Second\;Quadrant,\\ -\frac{\epsilon HR}{rd}\cdot \left(\alpha_{1}+\alpha_{2}\right) & , & Third\;Quadrant,\\ -\frac{\epsilon HR}{rd}\cdot \left(\alpha_{1}-\alpha_{2}\right) & , & Fourth\;Quadrant, \end{array}\right.$$

(8) $$\Delta C_{24}=\left\{\begin{array}{ccc} \frac{\epsilon HR}{rd}\cdot \left(\alpha_{1}-\alpha_{2}\right) & , & First\;Quadrant,\\ \frac{\epsilon HR}{rd}\cdot \left(\alpha_{1}+\alpha_{2}\right) & , & Second\;Quadrant,\\ -\frac{\epsilon HR}{rd}\cdot \left(\alpha_{1}-\alpha_{2}\right) & , & Third\;Quadrant,\\ -\frac{\epsilon HR}{rd}\cdot \left(\alpha_{1}+\alpha_{2}\right) & , & Fourth\;Quadrant. \end{array}\right.$$

To simplify the next calculation process, we can write Equations (7) and (8) as a matrix $\lambda_{\Delta C}$($\frac{\epsilon HR}{rd}=r$):

(9) $$\lambda_{\Delta C}=\left[\begin{array}{cccc} r(\alpha_{1}+\alpha_{2}) & r\left(\alpha_{1}-\alpha_{2}\right) & -r\left(\alpha_{1}+\alpha_{2}\right) & -r\left(\alpha_{1}-\alpha_{2}\right)\\ r\left(\alpha_{1}-\alpha_{2}\right) & r\left(\alpha_{1}+\alpha_{2}\right) & -r\left(\alpha_{1}-\alpha_{2}\right) & -r\left(\alpha_{1}+\alpha_{2}\right)\\ -r\left(\alpha_{1}+\alpha_{2}\right) & -r\left(\alpha_{1}-\alpha_{2}\right) & r\left(\alpha_{1}+\alpha_{2}\right) & r\left(\alpha_{1}-\alpha_{2}\right)\\ -r\left(\alpha_{1}-\alpha_{2}\right) & -r\left(\alpha_{1}+\alpha_{2}\right) & r\left(\alpha_{1}-\alpha_{2}\right) & r\left(\alpha_{1}+\alpha_{2}\right) \end{array}\right].$$

Then, the impedance expression of Equation (9) can be written as (k=jωr):

(10) $$\lambda =\left[\begin{array}{cccc} \frac{1}{k(\alpha_{1}+\alpha_{2})} & \frac{1}{k(\alpha_{1}-\alpha_{2})} & -\frac{1}{k(\alpha_{1}+\alpha_{2})} & -\frac{1}{k(\alpha_{1}-\alpha_{2})}\\ \frac{1}{k(\alpha_{1}-\alpha_{2})} & \frac{1}{k(\alpha_{1}+\alpha_{2})} & -\frac{1}{k(\alpha_{1}-\alpha_{2})} & -\frac{1}{k(\alpha_{1}+\alpha_{2})}\\ -\frac{1}{k(\alpha_{1}+\alpha_{2})} & -\frac{1}{k(\alpha_{1}-\alpha_{2})} & \frac{1}{k(\alpha_{1}+\alpha_{2})} & \frac{1}{k(\alpha_{1}-\alpha_{2})}\\ -\frac{1}{k(\alpha_{1}-\alpha_{2})} & -\frac{1}{k(\alpha_{1}+\alpha_{2})} & \frac{1}{k(\alpha_{1}-\alpha_{2})} & \frac{1}{k(\alpha_{1}+\alpha_{2})} \end{array}\right].$$

### 2.3. 2-DOF Angular Displacement Signal Decoupling Method

Equation (10) shows that $\alpha_{1}$ and $\alpha_{2}$ are coupled in $\Delta C_{13}$, $\Delta C_{24}$, and we cannot get $\alpha_{1}$ and $\alpha_{2}$ directly by detecting $\Delta C_{13}$ and $\Delta C_{24}$. Therefore, we need to design a signal processing circuit according to Equation (10), which can output a set of excitation signals. Driven by the excitation signal output from the signal processing circuit, the angular displacement signal components $\alpha_{1}$ and $\alpha_{2}$ coupled in $\Delta C_{13}$ and $\Delta C_{24}$ will be converted into two voltage signals $V_{1}sin\left(\omega_{1}t\right)$ and $V_{2}sin\left(\omega_{2}t\right)$ with different frequencies.

The voltage excitation signal can be written as a matrix δ($\omega_{1}\neq \omega_{2}$):

(11) $$\delta =\left[\begin{array}{c} A\sin\left(\omega_{1}t\right)+A\sin\left(\omega_{2}t\right)\\ A\sin\left(\omega_{1}t\right)-A\sin\left(\omega_{2}t\right)\\ -A\sin\left(\omega_{1}t\right)-A\sin\left(\omega_{2}t\right)\\ -A\sin\left(\omega_{1}t\right)+A\sin\left(\omega_{2}t\right) \end{array}\right]$$

where *A* is the amplitude of the two sinusoidal excitation signals, and $\omega_{1}t$ and $\omega_{2}t$ are the angular frequencies of two sinusoidal excitation signals.

(12) $$\lambda \cdot \delta =I_{Sig}$$

The current signal $I_{Sig}$ output by the capacitive sensor, under the excitation of the excitation signal, is:

(13) $$I_{Sig}=\left[\begin{array}{c} I_{1}\sin\left(\omega_{1}t\right)+I_{2}\sin\left(\omega_{2}t\right)\\ I_{1}\sin\left(\omega_{1}t\right)-I_{2}\sin\left(\omega_{2}t\right)\\ -I_{1}\sin\left(\omega_{1}t\right)-I_{2}\sin\left(\omega_{2}t\right)\\ -I_{1}\sin\left(\omega_{1}t\right)+I_{2}\sin\left(\omega_{2}t\right) \end{array}\right].$$

The current signal $I_{Sig}$ can be converted into the voltage signal $V_{Sig}$ by a transconductance amplifier circuit to realize the I/V convert ($V_{1}=4AG\alpha_{1}\omega j$, $V_{2}=4AG\alpha_{2}\omega j$, where *G* is the gain in the transconductance amplifier circuit).

(14) $$V_{Sig}=\left[\begin{array}{c} V_{1}\sin\left(\omega_{1}t\right)+V_{2}\sin\left(\omega_{2}t\right)\\ V_{1}\sin\left(\omega_{1}t\right)-V_{2}\sin\left(\omega_{2}t\right)\\ -V_{1}\sin\left(\omega_{1}t\right)-V_{2}\sin\left(\omega_{2}t\right)\\ -V_{1}\sin\left(\omega_{1}t\right)+V_{2}\sin\left(\omega_{2}t\right) \end{array}\right].$$

The i-th row of $V_{Sig}$ represents the voltage signal output by the capacitive sensor when the sensing electrode enters the i-th quadrant (i=1,2,3,4). Therefore, we can obtain $\alpha_{1}$ and $\alpha_{2}$ by demodulating $V_{Sig}$.

## 3. Simulation Setup

In order to verify the effectiveness of the capacitive sensor designed in Section 2, this chapter establishes a finite element model of the capacitive sensor based on the electromagnetic analysis software Maxwell, and performs a dynamic simulation on it according to the 2-DOF angular displacement signal decoupling method of the capacitive sensor, designed for the signal processing circuit. This is done through simulating the signal processing circuit to obtain the simulation curves, and making the simulation curves as a reference for designing the hardware circuit.

### 3.1. Finite Element Simulation and Analysis

**Figure 2:**
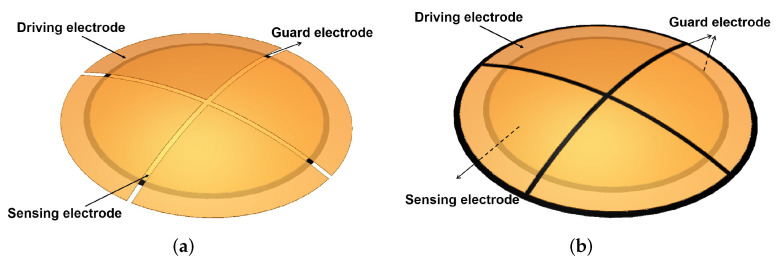
Simulation model of the capacitive sensor: (**a**) simulation Model 1 without shield electrode, (**b**) simulation Model 2 with a shield electrode.

To verify the differential output capacitance of the capacitive sensor shown in Equation (9), we, according to the parameters listed in Table 1, established the 3D finite element simulation model shown in Figure 2. Applying a guard ring to the sensing electrode effectively reduces the influence of the edge effect on the measurement results [16,17]. Therefore, this section mainly compares and analyzes the influence of applying a shield electrode to the driving electrode on the measurement results.

Consider the following case, that the sensing electrode moves in the directions $45^{\circ }$ and $60^{\circ }$, respectively, and analyze the relationship between the output capacitance of the capacitive sensor and the 2-DOF angular displacement of the sensing electrode. In the simulation, the range of the 2-DOF angular displacement is set to be [−4${}^{\circ }$, 4${}^{\circ }$], and the step is $0.1^{\circ }$.

In this study, the differential output capacitances $\Delta C_{13}$ and $\Delta C_{24}$ of the capacitive sensors is our analysis target. Therefore, we only simulate the differential output capacitance of the capacitive sensor and compare it with the theoretical values $\Delta C_{T13}$, $\Delta C_{T24}$ to verify the effectiveness of the capacitive sensor.

**Figure 3:**
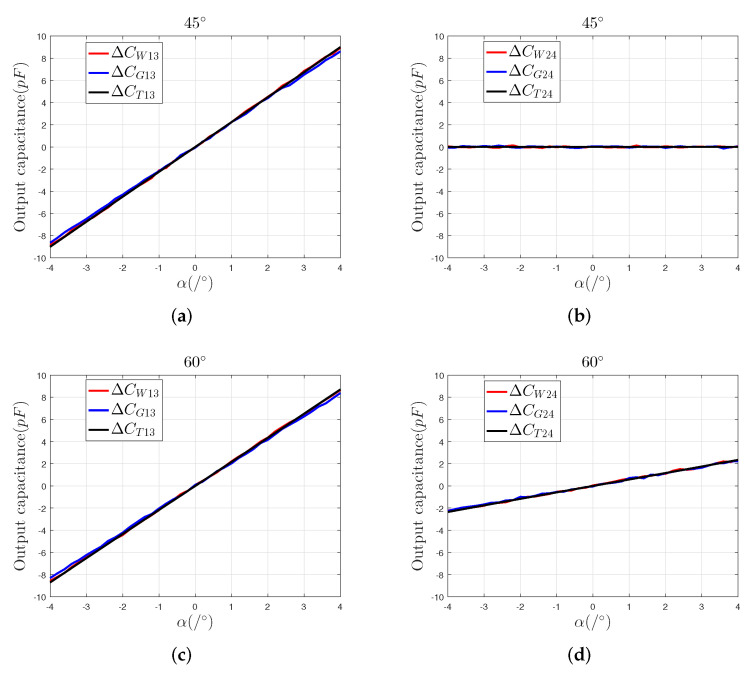
Simulation results: (**a**) the simulation output $\Delta C_{13}$ of the 3D finite element model in the direction of $45^{\circ }$, (**b**) the simulation output $\Delta C_{24}$ of the 3D finite element model in the direction of $45^{\circ }$, (**c**) the simulation output $\Delta C_{13}$ of the 3D finite element model in the direction of $60^{\circ }$, (**d**) the simulation output $\Delta C_{13}$ of the 3D finite element model in the direction of $60^{\circ }$.

As shown in Figure 3, the simulation curves of the two models are consistent with the trends of the theoretical curves, which can prove the feasibility and effectiveness of the the difference output capacitance calculation method proposed in Section 2. Compared with the simulation results of Model 2, the simulation results of Model 1 are closer to the theoretical results. This shows that applying shield electrodes to the drive electrodes may introduce an additional capacitance, which affects the system’s measurement accuracy. Therefore, Model 1 is more suitable as a template for the actual electrodes.

To verify the decoupling method in Section 2, we write the simulation results shown in Figure 3 as a matrix shown in Equation (9). According to the decoupling operation method shown in Equation (12), we obtained the simulated results and compared the simulation ones with the theoretical data to verify the decoupling method.

The $\alpha_{Ti}$, $\alpha_{si}^{1}$ and $\alpha_{si}^{2}$ in the curve diagram denote the theoretical output values, simulation Model 1 output values, and simulation Model 2 output values.

**Figure 4:**
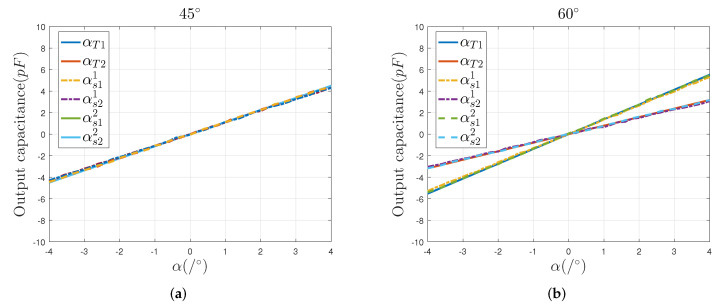
Comparison of theoretical and simulation values of the output capacitance: (**a**) the output capacitance in the direction of $45^{\circ }$, (**b**) the output capacitance in the direction of $60^{\circ }$.

As shown in Figure 4, the simulation results change linearly with the sensing electrode’s 2-DOF angular displacement in the directions of $45^{\circ }$ and $60^{\circ }$, respectively. In addition, there is almost no relative offset between the simulated and theoretical curves. Those simulation results show that the capacitive sensor, with a four-quadrant differential setup, can effectively eliminate the additional capacitance caused by the edge effect of the single electrode and can effectively improve the measurement accuracy and linearity of the sensor. Besides, the mathematical model established in Section 2 is correct and stable, which can be used to detect the 2-DOF angular displacement of the spherical pair.

### 3.2. Signal Processing Circuit Simulation

The above simulation results show that the decoupling method shown in Equation (12) is accurate, so this part is based on Equation (11) to design a signal processing circuit to decouple the 2-DOF angular displacement signal.

**Figure 5:**
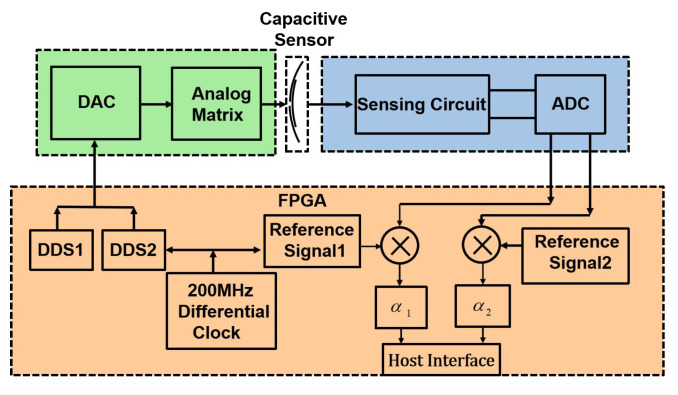
Functional block diagram.

As shown in Figure 5, the signal processing circuit consists of three parts: the driving circuit, the sensing circuit, and the field-programmable gate array (FPGA) digital circuit. The driving circuit comprises a digital-to-analog converter (DAC) and an operational amplifier network. The sensing circuit includes a TIA amplifier and an analog-to-digital converter (ADC). The FPGA digital circuit comprises two Direct Digital Synthesis (DDS) modules and an asynchronous demodulation module.

The two DDS modules generate two sinusoidal excitation signals $A\sin(\omega_{1}t)$ and $A\sin(\omega_{2}t)$, which are converted to the excitation matrix δ by the operational amplifier network. Under the excitation of δ, the capacitive sensor outputs a current signal $I_{Sig}$ to realize C/I. The sensing circuit senses the output current signal from the sensor and converts it into the voltage signal matrix $V_{Sig}$ to realize the decoupled operation shown in Equation (12).

**Figure 6:**
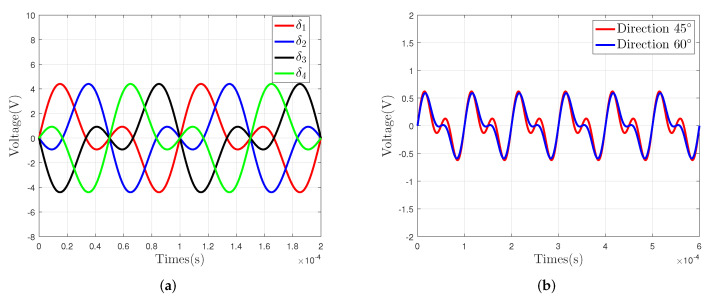
Simulation results of the signal process circuit: (**a**) driving circuit and (**b**) sensing circuit.

During the simulation experiment, we set the sensing electrode to move in the directions of $45^{\circ }$ and $60^{\circ }$, respectively, and made the DDS module output two sinusoidal signals. One signal has an amplitude of 2.5 V and a frequency of 10 kHz, and the other signal has an amplitude of 2.5 V and a frequency of 20 kHz. Then, we plotted the simulation curves of the driving circuit and sensing circuit in Figure 6. The simulation curve of δ is shown in Figure 6a, and the simulation curve of $V_{Sig}$ is shown in Figure 6b.

In this chapter, a 3D finite element model was established and a signal processing circuit was designed according to the mathematical model of the capacitive sensor in Section 2. The simulation results of the 3D finite element model and signal processing verified the mathematical model. Both of them can be used to guide the design and manufacture of the hardware circuit.

## 4. Experimental Setup

In this chapter, based on the finite element model of the capacitive sensor and signal processing circuit in Section 3, we designed and established the experimental device and the hardware circuit.

The 2-DOF angular displacement of the spherical pair can be regarded as a combination of 2-DOF angular displacements. To verify and analyze its measurement results, we decomposed the spherical pair’s 2-DOF angular displacement into a single DOF angular displacement of the azimuth axis of the driving electrode and a single DOF angular displacement of the pitch axis of the sensing electrode.

**Figure 7:**
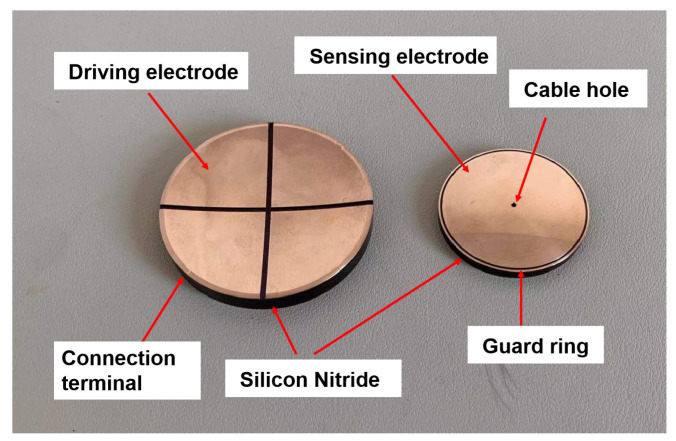
Capacitive sensor electrodes.

According to the Maxwell model simulation results, the driving electrodes and the sensing electrodes are constructed concerning the structure of simulation Model 1. The electrodes are shown in Figure 7, and their dimensions are listed in Table 2. The sensing and driving electrodes select silicon nitride ($Si_{3}N_{4}$) as the insulating substrate. In order to obtain the required positional accuracy of the insulated band of the driving electrode and guard ring of the sensing electrode, the substrates are manufactured by a precision CNC machining centre, and substrates will undergo a precision grinding procedure to achieve an excellent spherical surface accuracy. The electrodes are constructed by electroplating copper on the surface of the insulating substrate. The driving electrode is divided into four quadrants by a 4-mm wide insulated band. The four-quadrant parts, and the copper film on the convex surface of the sensing electrode, form a four-quadrant differential capacitive sensor.

**Figure 8:**
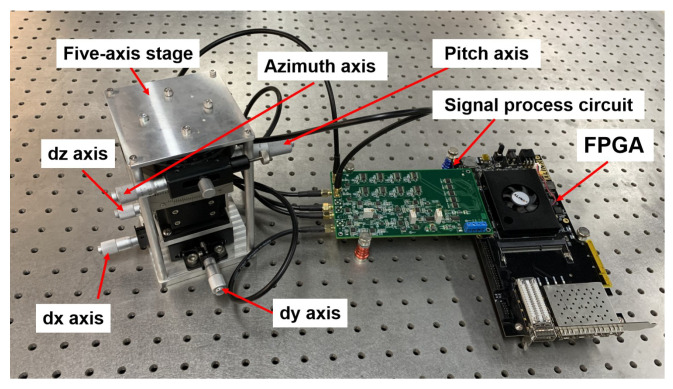
Capacitive sensor system.

As is shown in Figure 8, the experimental device comprises a five-axis stage, a signal processing circuit, and an FPGA digital circuit. The five-axis stage consists of two angular displacement axes (the azimuth axis and the pitch axis), three displacement axes (the dx axis, the dy axis, and the dz axis), and two support plates (the upper support plate and the down support plate), where the maximum angular resolution of the angular displacement axis is $0.1^{\circ }$, and the maximum resolution of the displacement axis is 0.01 mm, as well as the upper support plate, which is used for the installation of the driving electrode, and the down support plate, which is used for the installation of the sensing electrode. The azimuth angular displacement axis controls the upper support plate. The down support plate is jointly controlled by the dx displacement axis, the dy displacement axis, the dz displacement axis, and the pitch angular displacement axis.During the electrode installation process, we fix the sensing electrode and driving electrode on the down support plate and the upper support plate, respectively, and place a 1-mm thick spherical shape sheet on the sensing surface. Then, we install the down support plate and the upper support plate on the five-axis turntable in sequence, adjusting the dz axis to make the sensing electrode, the spherical shape sheet, and the driving electrode fully fit and record the dz axis position $z_{0}$. Finally, we adjust the dz axis to move the sensing electrode down to where the spherical shape sheet can be taken out. After removing the gasket, we tune the dz axis to reset the sensing electrode to position $z_{0}$ to make a 1-mm air gap between the sensing electrode and the driving electrode. The precise alignment of the driving electrode and the sensing electrode was achieved by fine-tuning the dx and dy axis of the five-axis stage to make the initial capacitances of the difference four-quadrant electrodes equal.

During the experiment, we can tune the azimuth axis to control the angular displacement direction of the sensing electrode, and the pitch axis to control the angular displacement of the sensing electrode. The jointly controlled axes can enable the sensing electrode to rotate, relative to the driving electrode, in any direction.

## 5. Measurement Experiments

In this section, we perform measurement experiments based on the above sensor prototype and the signal processing circuit. We use the simulation results in Section 3 as a reference during the analysis of the experimental results.

### 5.1. Signal Processing Circuit

**Figure 9:**
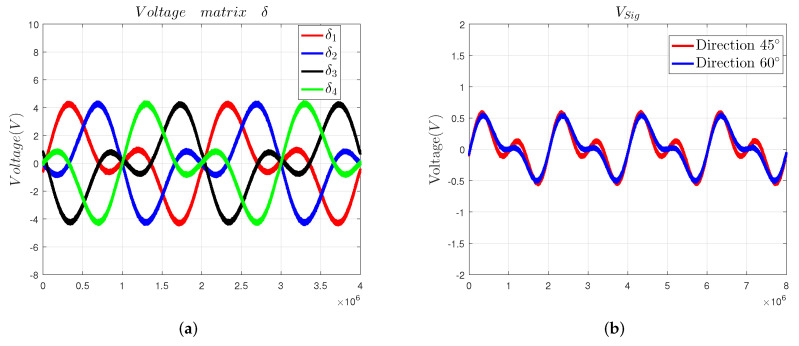
The actual waveform of the signal process circuit: (**a**) the voltage matrix δ and (**b**) the voltage signal $V_{sig}$.

First, we verify the signal processing circuit and plot the output curves of the driving circuit in Figure 9a, and the output curves of the voltage signal $V_{sig}$ in Figure 9b.

By comparing the curves shown in Figure 5 and Figure 9, we can know that the theoretical output curves of the signal processing circuit are consistent with the simulation output curves. Those curves indicate that the designed signal processing can be used to detect the 2-DOF angular displacement signal of the sensor prototype.

### 5.2. Angular Displacement Measurement

We turn the five-axis platform to rotate the sensing electrodes in the directions of $45^{\circ }$ and $60^{\circ }$ and can perform measurement experiments to study the change in the differential output capacitance of the capacitive sensor. During the whole experiment process, the variation range of the pitch angular displacement component α is still [−$4^{\circ }$, $4^{\circ }$], and the variation rate is $0.1^{\circ }$/step.

**Figure 10:**
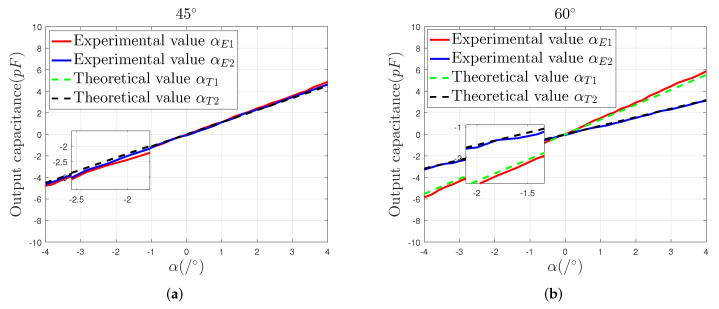
Comparison of experimental and simulation values of the output capacitance: (**a**) the output capacitance in the direction of $45^{\circ }$, (**b**) the output capacitance in the direction of $60^{\circ }$.

A comparison of the actual measured output capacitance and theoretical output capacitance of the sensor prototype, as the sensing electrode moves in the directions of $45^{\circ }$ and $60^{\circ }$, respectively, is shown in Figure 10. As well as keeping consistent with the variation trend of the simulation curves, the measured output capacitance curves are similarly symmetrically distributed on both sides of the reference line (α = 0), showing that the four-quadrant differential electrode setup has good mutual position accuracy after being assembled.

It also can be seen from Figure 10 that, compared with the solution value of $\alpha_{1}$, the solution value of $\alpha_{2}$ is closer to the theoretical data. In the $45^{\circ }$ direction, the maximum measurement error of $\alpha_{1}$ is 0.39 pF, and the maximum measurement error of $\alpha_{2}$ is 0.18 pF. In the $60^{\circ }$ direction, the maximum measurement error of $\alpha_{1}$ is 0.38 pF, and the maximum error of $\alpha_{2}$ is 0.13 pF. In different directions, the deviation between the slope of the $\alpha_{E1}$ curve and the theoretical curve slope is slightly larger than that of $\alpha_{E2}$. That is because, during the decoupling calculation process shown in Equation (12), the solution of $\alpha_{2}$ is realized by the differential calculations $\Delta C_{13}$ and $\Delta C_{24}$, which can effectively suppress the common mode noise, for example, the assembly error of the sensor prototype and the noise of the signal processing circuit.

Therefore, compared with $\alpha_{E2}$, $\alpha_{E1}$ will be added to a part of the common-mode noise $N_{com}$. To reduce the influence of $N_{com}$ on $\alpha_{E1}$, first, we calculate the ratio $N_{1}=\left(K1/S1\right)$ of the slope K1 of the curve of ae1 to the slope S1 of the theoretical curve, and the ratio of the slope K2 of the curve of $\alpha_{E2}$ to the slope of the theoretical curve $N_{2}=\left(K2/S2\right)$. Then, we use the term $M=N_{1}/N_{2}$ to estimate the effect of $N_{com}$ on $\alpha_{E1}$. In the $45^{\circ }$ direction, M≈1.03. In the $60^{\circ }$ direction, M≈1.06. Therefore, we make the intermediate value of $N_{1}$ and $N_{2}$ be *N*, and let N=1.05. Finally, to eliminate the influence of $N_{com}$ on the slope of $\alpha_{E1}$, we compensate for $\alpha_{E1}$ through the operation of $\alpha_{E1}/M$.

**Figure 11:**
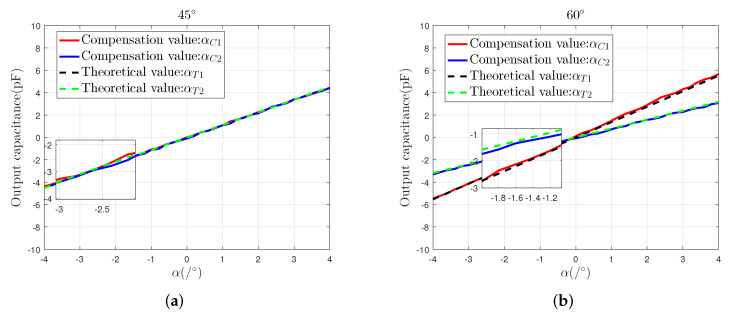
Compensated results: (**a**) the compensated results in the direction of $45^{\circ }$, (**b**) the compensated results in the direction of $60^{\circ }$.

It can be seen from Figure 11 that, in the direction of $45^{\circ }$, the maximum measurement error of $\alpha_{C1}$ decreases from 0.39 pF to 0.28 pF after compensation. In the direction of $60^{\circ }$, the maximum measurement error of $\alpha_{C1}$ decreases from 0.38 pF to 0.17 pF after compensation. In different directions, compared with the uncompensated $\alpha_{E1}$ curve, the compensated $\alpha_{C1}$ curve is closer to the theoretical curve. This shows that the influence of common-mode noise $N_{com}$ on the measurement result of $\alpha_{E1}$ can be reduced through compensation.

**Figure 12:**
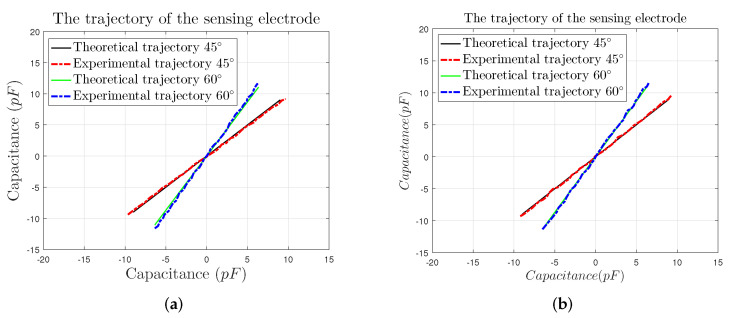
The trajectory of the sensing electrode: (**a**) the trajectories derived by uncompensated experimental values, (**b**) the trajectories derived by compensated experimental values.

By taking the decoupled output capacitance of the sensor prototype as a dependent variable and variable, respectively, the curves, which can also be seen as the trajectory of the sensing electrode, are plotted in Figure 12 [18].

From Figure 11, we can know that the trajectories obtained from the measured values $\alpha_{E1}$ and $\alpha_{E2}$ coincide with the theoretical trajectories, which are in good agreement. The trajectory obtained by the compensation values $\alpha_{C1}$ and $\alpha_{C2}$ is closer to the theoretical trajectory.

This chapter developed a spherical pair capacitive sensor with a differential four-quadrant electrode setup and a signal processing circuit. Their feasibility and effectiveness were verified by actual measurements, which indicated that the measurement system, with the above components as the main body, can simultaneously measure 2-DOF angular displacement on a spherical pair.

## 6. Conclusions

This study presents a new method for measuring a spherical pair’s two-degrees-of-freedom (2-DOF) angular displacement using the proposed new spherical capacitive sensor with a four-quadrant differential electrode setup. The proposed spherical capacitive sensor has an integrated structure that occupies a smaller space and can be easily integrated into a spherical pair, compared to the existing angular displacement detection method. The mathematical models for detecting the 2-DOF angular displacement of the spherical pair are established. An experiment device constituted mainly by the sensor and signal processing circuit is tested. The details may be summarized as follows:

- Simulation and experimental results show that the mathematical model established, based on the differential four-quadrant electrode structure, can accurately predict the variation relationship between the 2-DOF angular displacement signal of the sensing electrode and the differential output capacitance;
- The signal processing circuit can decouple the components $\alpha_{1}$ and $\alpha_{1}$ of the 2-DOF angular displacement signal coupled in the differential capacitance signal into two voltage signals with different frequencies;
- Experimental results show that within the 2-DOF angular displacement range of $\pm 4^{\circ }$, the results measured by the designed sensor system are up to 89.7% of the theoretical results. In addition, the influence of common-mode noise can be effectively reduced through numerical compensation;
- The spherical capacitive sensor proposed in this study has an integrated structure. It can be integrated into the spherical pair, effectively avoiding the influence of accumulated installation errors on measurement accuracy. That is important for promoting the application of the spherical pair in aviation optoelectronic pods.

## Figures and Tables

**Table 1:** Simulation model parameters of the capacitive sensor.

**Driving Electrode**	**Symbol**	**Value**	**Unit**
The curvature radius	$R_{D}$	127	mm
The projection circle radius	$r_{D}$	50	mm
Thickness	$T_{D}$	1	um
**Sensing Electrode**	**Symbol**	**Value**	**Unit**
Start angle	$\alpha_{start}$	−4	$degree\;{(}^{\circ })$
Stop angle	$\alpha_{stop}$	4	$degree\;{(}^{\circ })$
The curvature radius	*R*	126	mm
The projection circle radius	*r*	40	mm
Thickness	*T*	1	um

**Table 2:** Dimensions of parameters of the prototype sensor.

Spherical Electrode Dimensional Parameters	Dimension
The curvature radius of the driving electrode ($R_{D}$)	127 mm
The curvature radius of the sensing electrode (*R*)	126 mm
The projection circle radius of the driving electrode ($r_{D}$)	50 mm
The projection circle radius of the sensing electrode (*r*)	40 mm
The gap between the driving electrode and sensing electrode (*d*)	1 mm

## Data Availability

Not applicable.

## References

[1] Leninger B. (2008). Autonomous real-time ground ubiquitous surveillance-imaging system (ARGUS-IS). Proc. Spie Int. Soc. Opt. Eng..

[2] Garner H. Design and analysis of an absolute non-contact orientation sensor for wrist motion control. Proceedings of the 2001 IEEE/ASME International Conference on Advanced Intelligent Mechatronics.

[3] Lee K.M., Zhou D. (2004). A real-time optical sensor for simultaneous measurement of three-DOF motions. IEEE/ASME Trans. Mech..

[4] Lim C.K. (2011). A novel approach for positional sensing of a spherical geometry. Sens. Actuators A Phys..

[5] Wang W. (2003). Design and Control of a Novel Spherical Permanent Magnet Actuator with Three Degrees of Freedom. IEEE/ASME Trans. Mechatron..

[6] Foong S. (2012). Harnessing Embedded Magnetic Fields for Angular Sensing With Nanodegree Accuracy. IEEE/ASME Trans. Mechatron..

[7] Yang W. (2017). Simulation and analysis of influence of magnetic field distribution in intelligent ball hinge. China Meas. Test.

[8] Hu P. (2018). Optimized design and accuracy improvement of intelligent ball hinge. Chin. J. Sci. Instrum..

[9] Hu P. (2019). A New Modeling Method of Angle Measurement for Intelligent Ball Joint Based on BP-RBF Algorithm. Appl. Sci..

[10] Liao P. (2018). Measurement of the Precision Spatial Rotation Angle of Ball Joint Based on Artificial Neural Network. Master’s Thesis.

[11] Hu P. (2018). Measurement method of rotation angle and clearance in intelligent spherical hinge. Meas. Sci. Technol..

[12] Hu P. (2020). A New Method for Measuring the Rotational Angles of a Precision Spherical Joint Using Eddy Current Sensors. Sensors.

[13] Wang W. (2019). A Novel Approach for Detecting Rotational Angles of a Precision Spherical Joint Based on a Capacitive Sensor. Micromachines.

[14] Zou M. (2021). Fiber-Tip Polymer Clamped-Beam Probe for High-Sensitivity Nanoforce Measurements.

[15] Geng Z. (2021). Review of Geometric Error Measurement and Compensation Techniques of Ultra-Precision Machine Tools.

[16] Ahn H.-J. (2005). Nonlinear analysis of cylindrical capacitive sensor. Meas. Sci. Technol..

[17] LI X. (2000). The influence of electric-field bending on the nonlinearity of capacitive sensors. IEEE Trans. Instrum. Meas..

[18] Kui X. (2017). A T-Type Capacitive Sensor Capable of Measuring5-DOF Error Motions of Precision Spindles. Sensors.

